# Thermal Molding of Organic Thin-Film Transistor Arrays on Curved Surfaces

**DOI:** 10.1186/s11671-017-2113-x

**Published:** 2017-05-12

**Authors:** Masatoshi Sakai, Kento Watanabe, Hiroto Ishimine, Yugo Okada, Hiroshi Yamauchi, Yuichi Sadamitsu, Kazuhiro Kudo

**Affiliations:** 1 Department of Electrical and Electronic Engineering, Chiba University, 1-33 Yayoi-cho, Inage-ku, Chiba, 263-8522 Japan; 2 Center for Innovative Research Nippon Kayaku Co., Ltd. 3-31-12 Shimo, Kita-ku, Tokyo, 115-8588 Japan

**Keywords:** Flexible electronics, Organic semiconductor, Strain, Curved surface, Artificial tactile sense, C _8_-BTBT, Thin-film transistor

## Abstract

In this work, a thermal molding technique is proposed for the fabrication of plastic electronics on curved surfaces, enabling the preparation of plastic films with freely designed shapes. The induced strain distribution observed in poly(ethylene naphthalate) films when planar sheets were deformed into hemispherical surfaces clearly indicated that natural thermal contraction played an important role in the formation of the curved surface. A fingertip-shaped organic thin-film transistor array molded from a real human finger was fabricated, and slight deformation induced by touching an object was detected from the drain current response. This type of device will lead to the development of robot fingers equipped with a sensitive tactile sense for precision work such as palpation or surgery.

## Background

Plastic and printed electronics have recently attracted extensive interest for flexible and stretchable device applications. However, research on device fabrication technologies for curved surfaces is not as active even though many modern commodities consist of smoothly curved plastics such as poly(ethylene terephthalate) bottles, blister packs, medical tubes, and connectors. Although the adaptability of stretchable electronics to spherical surfaces has been demonstrated [1], stretchable devices generally cannot maintain their self-standing shape on their own. Therefore, not only flexible or stretchable devices but also curved-surface devices are needed for a wide range of applications. For example, if robot fingertips could be equipped with sensitive thermal and tactile sense using curved-surface electronic devices, more precise surgical operation could be performed using medical robots.

In this paper, we present a novel technique to fabricate an electronic device array on a freely designed curved surface via thermal molding of plastic sheets and direct melting of organic semiconductors with subsequent recrystallization, which is expected to enable the preparation of 3D plastic electronics. We fabricated a fingertip-shaped organic thin-film transistor (OTFT) array as a curved surface device test case and succeeded in detecting slight deformation induced by a soft touch from an object.

## Methods

**Fig. 1:**
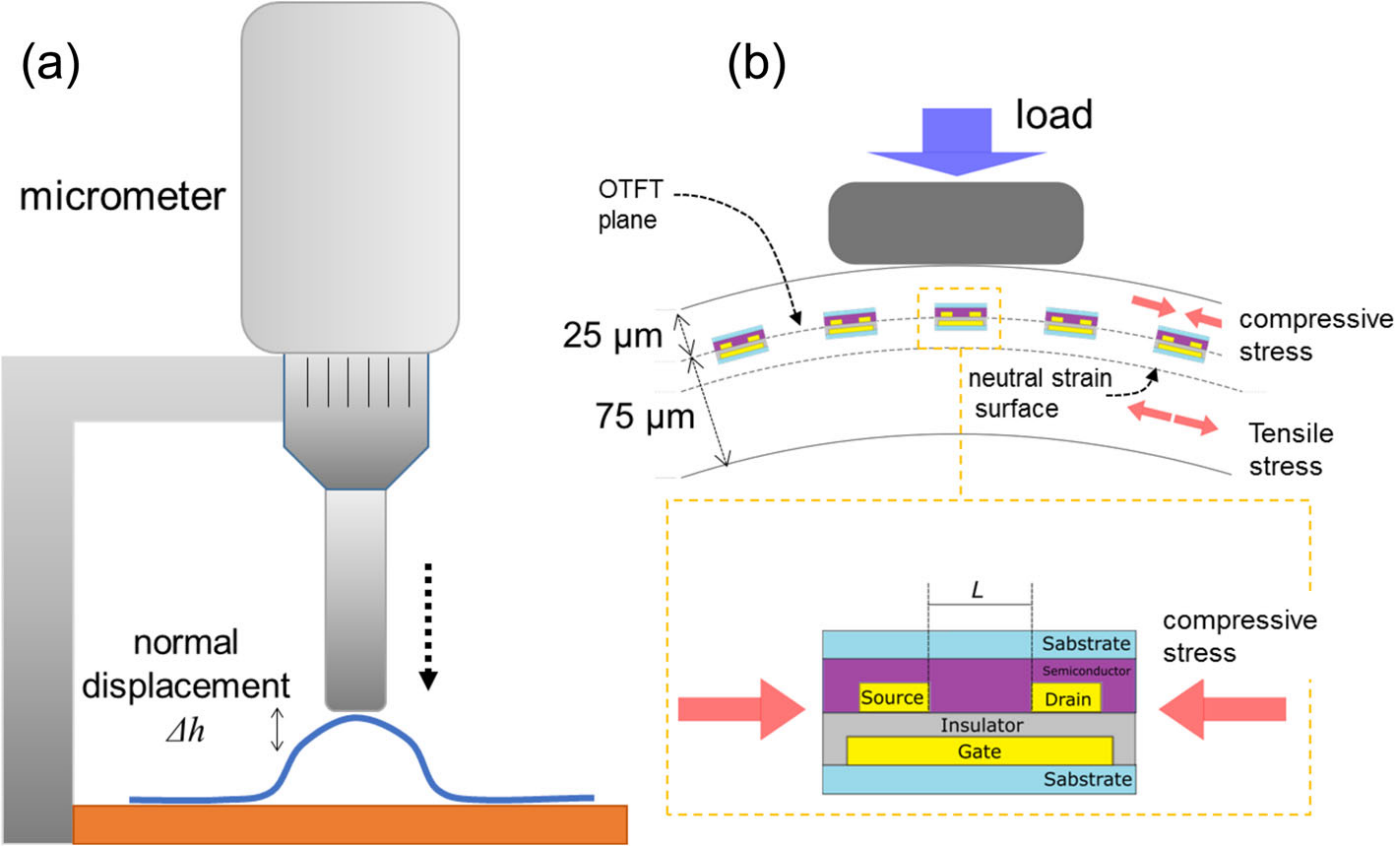
**a** Schematic illustration of the tactile sensitivity tester, which consisted of a micrometer and sample stage. The sample was pressed by the rod of the micrometer in the normal direction. The normal displacement of *Δ* *h* is the distance from the initial top height of the fingertip-shaped surface to the height of the pressed surface. **b** Schematic principle of tactile sensing by OTFT array. Electronic devices placed on a neutral strain surface are not exposed to lateral strain because the tensile and compressive strain cancel each other on the neutral strain surface [27–31]. However, the OTFTs placed on the off-neutral strain surface were exposed to lateral compressive strain due to the slight distortion of the fingertip-shaped curved surface

Thin poly(ethylene naphthalate) (PEN) films (Teijin Ltd., Japan) with thicknesses of 75 *μ*m were used as substrates. The substrates were coated with a 900-nm-thick parylene-SR layer after thermal evaporation of a Au gate electrode pattern. The thick parylene layer was used to prevent stochastic gate leakage. A Au contact electrode pattern was then deposited on the surface and was chemically treated using a pentafluorobenzenethiol self-assembled monolayer (PFBT-SAM) to reduce the contact resistance [2–6]. An appropriate amount of dioctylbenzothienobenzothiophene (C _8_-BTBT) powder [7–22] was transferred onto the substrate, and then, another PEN film with a thickness of 25 *μ*m was placed on the powder. The pair of PEN films and organic powder were then thermally pressed using a mold to fabricate a thin-film device with curved surfaces. The mold temperature was gradually increased up to 170 °C, and then, a pressure of 85 kPa was applied. This state was maintained for 5 min under active control of the applied pressure. The temperature of the sample was gradually decreased to 40 °C, and then, the heat clamp was opened and the sample was retrieved. We modified a previously reported thermal pressing method [23] by replacing the press plate with a curved mold. The curved mold used in the study of strain induced during the formation of a spherical surface consisted of a pair of spherical lens, and the mold in the fingertip-shaped OTFT array was an original mold formed using the fingertip of one of the authors. The minimum radius of curvature of the original mold was approximately 7 mm, as estimated using a spherical approximation. Electrical measurements of the fabricated TFTs were performed using a source meter in the dark under vacuum (Keithley 6430 and 2635A). For tactile sensitivity tests, we constructed an original tester. The configuration of the tester and the principle of the measurement are shown in Fig. 1. The tester consisted of a micrometer and sample stage, on which electrical measurements were performed. The center of the fingertip-shaped OTFT array was weakly pressed and slightly distorted by the rod of the micrometer, and the increase or decrease of the drain current of the OTFT was measured. As illustrated in Fig. 1 b, simple normal compression induces lateral compressive strain in the OTFT because the OTFT plane was on the compression side of 25% from the neutral strain surface toward the total thickness. Flexible electronic devices are typically placed on a neutral strain surface to prevent irreversible degradation or a change of characteristics during bending. In our work, this standard practice was moderately ignored for our purposes.

## Results and Discussion

**Fig. 2:**
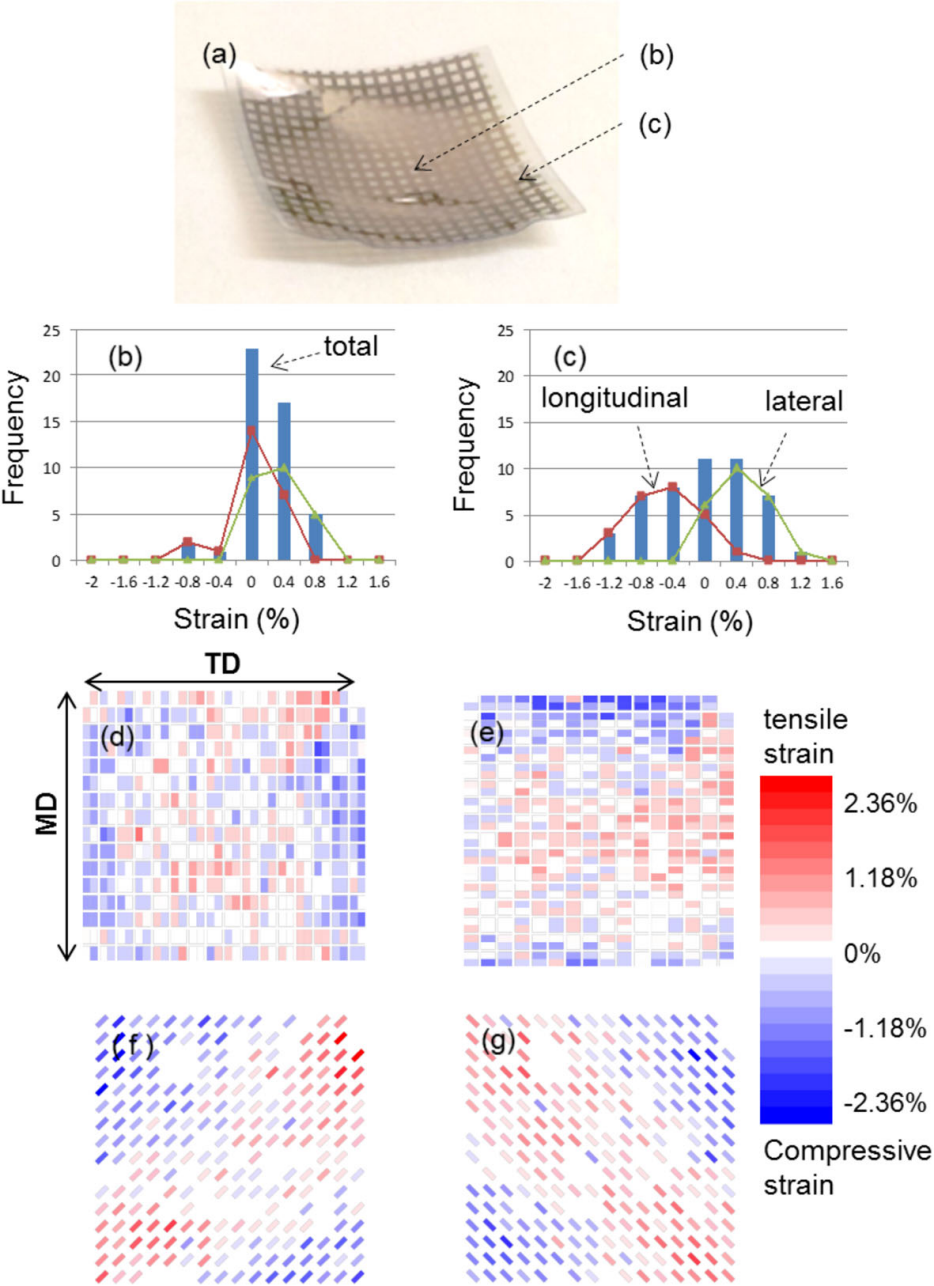
Estimation of tensile and compressive strain induced by forming spherical surface from planar sheet. **a** Photograph of spherical PEN surface with square pattern of Au thin film. Histograms of longitudinal and lateral side lengths of square pattern **b** at the center of the spherical surface and **c** in the peripheral region. Strain distribution map for **d** longitudinal length, **e** lateral length, and **f, g** diagonal length. MD denotes the machine direction, which is the direction in which the PEN film was drawn into the roll during the manufacturing process, and TD denotes the transverse direction, which is the direction perpendicular to MD in the plane of the film. The thermal contraction ratios along MD and TD are generally different for commercial plastic films

We first estimated the strain distribution of the plastic film substrate generated by the formation of the curved surface from the planar sheet. Even a flexible film cannot be completely attached to a general curved surface because the Ricci scalar (scalar curvature) of the planar sheet is zero even when the sheet is rolled into a cylinder. However, the Ricci scalar is nonzero for a general curved surface. For example, the Ricci scalar of a spherical surface is 2/ *r* ^2^, where *r* is the radius of the spherical surface. Two surfaces with different Ricci scalar values cannot be fitted with each other without causing wrinkles. In this work, a spherical lens with a radius of curvature of 25.8 mm was used as a mold because a spherical surface is the simplest curved surface; the radius of curvature is uniform for a spherical surface. The strain distribution was estimated by measuring the side and diagonal lengths of the square pattern of the Au thin film evaporated on the PEN films. A photograph of the spherical surface test piece is presented in Fig. 2 a. The spherical surface was precisely formed except for the four corners, which was expected because the four corners of the PEN films were not covered by the mold, i.e., the pair of convex and concave spherical lens. We compared the side and diagonal lengths of the square pattern before and after the formation of the spherical shape. Histograms of the side length are presented in Fig. 2 b, c. In Fig. 2 b, the longitudinal and lateral lengths of the square pattern have almost the same distribution at the center of the spherical surface; however, in Fig. 2 c, the distributions of the longitudinal and lateral lengths are clearly separated, and both tensile and compressive strain are observed in the peripheral region. Both the compressive and tensile strain increased from the center to the peripheral regions. The length measurements are summarized in Fig. 2 d–g. These results indicate that the tensile and compressive strains occur in the radial and circumferential directions, respectively. The observed tensile strain is due to the stretching deformation of the PEN film resulting from the molding. However, the compressive strain is possibly caused by natural thermal contraction of the PEN film, which is effective for the formation of a curved surface device without causing excess stretching of the substrate film. In addition, in this case, the Poisson effect occurs in the thickness direction because the plastic sheet was originally fabricated by stretching in the plane direction; therefore, the sheet shrinks in the in-plane direction and expands in the normal direction to approach the original dimensions.

**Fig. 3:**
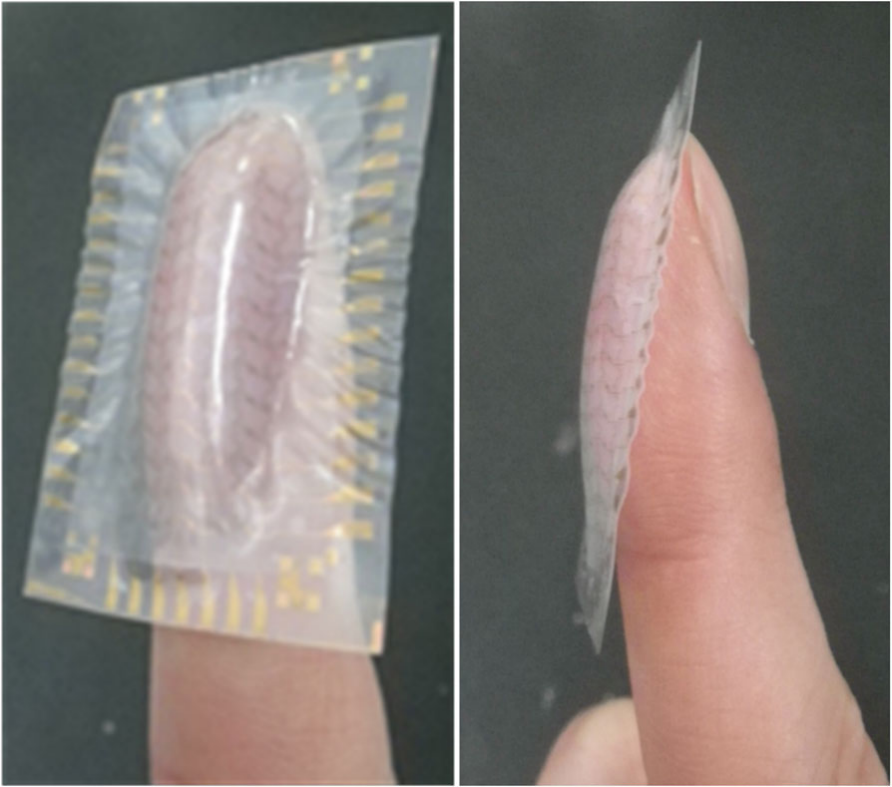
Photograph of fingertip-shaped OTFT array on the human finger

Figure 3 presents a photograph of the fingertip-shaped plastic OTFT array fabricated using our thermal molding technique. There are 88 OTFTs on the fingertip-shaped surface, and measurement terminals were arranged along the periphery in the planar region. Some wrinkles are observed in the peripheral planar region; however, the curved surface region was precisely formed along the mold shape. Therefore, this device could be completely attached onto a human finger, as shown in Fig. 3. The interval of the OTFT array was 1 mm, which is shorter than the separation distance at which a human finger can distinguish two point contacts by tactile sense. The channel lengths and widths of the individual OTFTs were 20 and 1340 *μ*m, respectively.

**Fig. 4:**
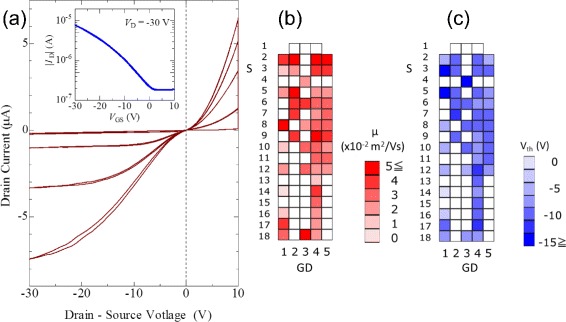
**a** Typical output and transfer characteristics of fingertip-shaped OTFT array. Maps of the observed **b** field-effect hole mobility and **c** threshold voltage in the OTFT array, respectively. The colored pixels indicate working OTFTs, and the empty pixels indicate poor FET characteristics

The typical TFT characteristics for the OTFT array are presented in Fig. 4 a. *p*-type OTFT characteristics were observed, reflecting the *p*-type semiconductor nature of the C _8_-BTBT semiconductor. Compared with the standard C _8_-BTBT OTFT characteristics observed for OTFTs fabricated using other solvent-free methods [23–26], the carrier injection barrier appears to be relatively high in the output characteristics. Although the Au contact electrodes were chemically treated with PFBT-SAM to reduce the contact resistance, PFBT-SAM is thermally unstable and gradually deteriorates above 150 °C. Our thermal molding process includes thermal processing at 170 °C for more than 10 min. Therefore, it is possible that the effect of the PFBT-SAM layer deteriorated because of the thermal history. The increase of the off current is possibly due to the relatively high thickness of the organic semiconductor layer. A thick semiconductor layer can cause a large off current in a TFT structure because C _8_-BTBT naturally exhibits high electrical conductivity. The large off current is not due to the gate leakage current because the observed gate leakage current was approximately 10 ^−12^ A, which is much smaller than the drain current. The estimated effective field-effect hole mobility was approximately 0.05 cm ^2^/V s. The relatively low effective mobility for C _8_-BTBT is mainly due to the short channel length of 20 *μ*m; the contact resistance generally dominates over the observed effective mobility for a short channel length [9].

The carrier mobility and threshold voltage map presented in Fig. 4 b, c summarizes the properties of the 88 TFTs. The working OTFTs are colored along the effective field-effect mobility. A group of working OTFTs are observed. However, the thicknesses of the OTFTs were not sufficiently uniform because of the limitation in mechanical precision of the present mold. Therefore, the distributions of the observed field-effect hole mobility and threshold voltage were wide at present.

**Fig. 5:**
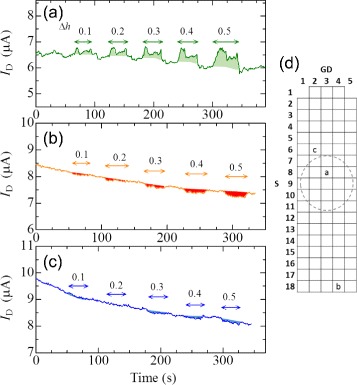
Time response of the drain current to a slight touch with an object. The variation of the drain current in an OTFT in the fingertip-shaped OTFT array was detected in relation to a vertical displacement of *Δ* *h* under *V*_GS_ = −30 *V* and *V*_DS_ = −30 *V*. The observed drain current in the OTFT increased during the application of the displacement, and the drain current difference during the compression clearly depends on *Δ* *h*. *d* Location map of the OTFT array and contact area with micrometer rod. The dotted circle indicates the direct contact area of the OTFT array with the micrometer rod. The (S8, GD3) pixel is directly under the rod, the (S18, GD4) pixel is far from the contact area, and the (S6, GD3) pixel is near the contact area

**Fig. 6:**
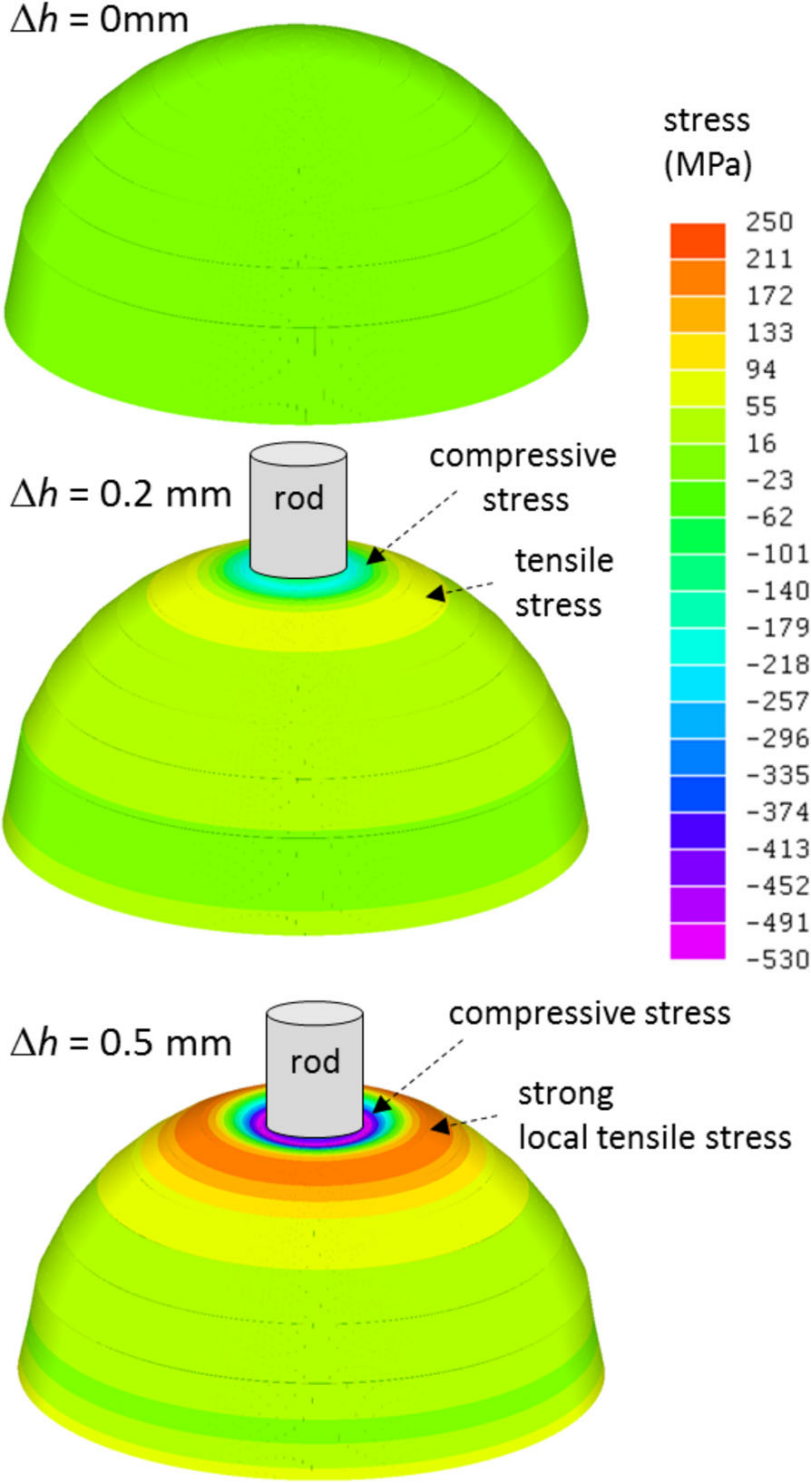
Calculated principal stress distribution on the modeled thin PEN hemisphere face calculated using finite element method. With increasing *Δ* *h*, compressive stress arises within and near the contact area, and strong local tensile stress arises in the periphery of the contact area

The fingertip-shaped OTFT array was configured for use as a robot finger for precision work such as palpation or surgery using a sensitive tactile sense. Therefore, we performed tactile sensitivity tests. The drain current of an OTFT was continuously monitored under the bias condition of *V*_DS_ = −30 *V* and *V*_GS_ = −30 *V*. It is possible to calibrate the sensitivity of each OTFT by adjusting the bias condition. However, the bias condition was fixed in the present study. The fingertip shape was placed on the test equipment and slightly pressed in the normal direction of *Δ* *h* from the initial height by the micrometer, as illustrated in Fig. 1 a. The initial drain current (*I*_D_) observed in the (S8, GD3) pixel in Fig. 5 d was approximately 6.5 *μ*A with no load, as shown in Fig. 5 a. During compression with normal displacement (*Δ* *h* of 0.1, 0.2, 0.3, 0.4 and 0.5 mm), the increase of *I*_D_ was approximately 0.3, 0.4, 0.5, 0.7 and 0.9 *μ*A, respectively. The increase of the drain current clearly depended on *Δ* *h*. These responses were reversible and highly sensitive. Therefore, OTFTs that are directly pressed by the micrometer rod received compressive stress, which agrees with both the simple prediction illustrated in Fig. 1 b and the calculated stress distribution simulated by the finite element method on a hemispherical surface shown in Fig. 6. On the other hand, because the entire shape was affected by the applied displacement, all the working OTFTs, including those that were not directly touched, also detected the displacement, as shown in Fig. 5 b, c. Figure 5 b shows that the (S18, GD4) pixel was obviously separated from the contact area with the micrometer rod. In Fig. 5 b, a decrease of *I*_D_ was observed during the application of displacement, which corresponds to the detection of tensile strain in the pixel. The decrease of *I*_D_ corresponds to the *Δ* *h* in Fig. 5 b. Moreover, Fig. 5 c shows the response observed slightly outside the rim of the micrometer rod. This region was unique. *I*_D_ clearly decreased with increasing *Δ* *h* in the 0.3–0.5 mm region; however, *I*_D_ slightly increased for *Δ* *h* of 0.1 mm, i.e., compressive stress was applied to the (S6, GD2) pixel during the initial stage of deformation, and the tensile stress gradually increased with increasing displacement. The mechanism for the generation of compressive stress during the initial stage is the same as that illustrated in Fig. 1 b; however, a hemispherical surface exhibits structural resistance against normal compression compared with the simple cylindrical surface assumed in Fig. 1 b. Therefore, the fingertip-shaped surface locally sunk at and near the contact area with the micrometer rod, as illustrated in Fig. 6 (*Δ* *h* = 0.5 mm) because our device sheet was very thin. Therefore, the (S6, GD2) pixel exhibited compressive stress during the initial stage of deformation, and tensile stress arose by the local sinking of the surface due to the external force transmitted by the rod. The surface of our real finger is also slightly and intricately distorted with a soft touch of an object, which enables detection of the hardness and texture of the object.

## Conclusions

The fabrication of plastic electronics on curved surfaces using thermal molding was demonstrated in this work. The induced strain distribution of PEN films when planar sheets were deformed into spherical surfaces clearly indicated that natural thermal contraction played an important role in the formation of the curved surface to avoid excess tensile strain, which could result in fracture of the electrode pattern. The OTFT characteristics of a fingertip-shaped OTFT array molded from a real human finger were also examined. The drain current response to slight deformation induced by touching an object clearly increased with increasing internal strain. By scanning the OTFTs, it would be possible to detect the hardness or edge of an object based on the internal strain distribution. This type of device array fabricated on a curved surface will enable the development of robot fingers equipped with a sensitive tactile sense, which is sufficient for precision work such as palpation or surgery.
